# Supplementary material for: Pyruvate Dehydrogenase Kinase 1 inhibition mediated oxidative phosphorylation enhancement in cartilage promotes osteoarthritis progression
Source: BMC Musculoskelet Disord. 2023 Jul 20;24:597. doi: 10.1186/s12891-023-06585-6 (PMC10357736; doi:10.1186/s12891-023-06585-6)
Supplement: Supplementary file 1 — Additional file 1. [file 12891_2023_6585_MOESM1_ESM.zip › BP.Gsea.1653623667859/GOBP_MYELOID_CELL_HOMEOSTASIS.html]

Details for gene set GOBP\_MYELOID\_CELL\_HOMEOSTASIS[GSEA]

|  || Dataset | 55457\_collapsed\_to\_symbols.cls.cls #OA\_versus\_normal.cls.cls #OA\_versus\_normal\_repos |
| Phenotype | cls.cls#OA\_versus\_normal\_repos |
| Upregulated in class | normal |
| GeneSet | GOBP\_MYELOID\_CELL\_HOMEOSTASIS |
| Enrichment Score (ES) | -0.3850119 |
| Normalized Enrichment Score (NES) | -1.7668176 |
| Nominal p-value | 0.0 |
| FDR q-value | 0.119665444 |
| FWER p-Value | 0.998 |
Table: GSEA Results Summary

  

Fig 1: Enrichment plot: GOBP\_MYELOID\_CELL\_HOMEOSTASIS      
 Profile of the Running ES Score & Positions of GeneSet Members on the Rank Ordered List

  

| SYMBOL | TITLE | RANK IN GENE LIST | RANK METRIC SCORE | RUNNING ES | CORE ENRICHMENT || 1 | GPI | glucose-6-phosphate isomerase [Source:HGNC Symbol;Acc:HGNC:4458] | 166 | 0.842 | 0.0042 | No |
| 2 | HOXB6 | homeobox B6 [Source:HGNC Symbol;Acc:HGNC:5117] | 269 | 0.774 | 0.0121 | No |
| 3 | ALAS1 | 5'-aminolevulinate synthase 1 [Source:HGNC Symbol;Acc:HGNC:396] | 288 | 0.757 | 0.0261 | No |
| 4 | KCNQ1 | potassium voltage-gated channel subfamily Q member 1 [Source:HGNC Symbol;Acc:HGNC:6294] | 391 | 0.709 | 0.0327 | No |
| 5 | MAEA | "macrophage erythroblast attacher, E3 ubiquitin ligase [Source:HGNC Symbol;Acc:HGNC:13731]" | 658 | 0.604 | 0.0241 | No |
| 6 | NCSTN | nicastrin [Source:HGNC Symbol;Acc:HGNC:17091] | 666 | 0.601 | 0.0359 | No |
| 7 | KAT7 | lysine acetyltransferase 7 [Source:HGNC Symbol;Acc:HGNC:17016] | 1125 | 0.491 | 0.0100 | No |
| 8 | DNASE2 | "deoxyribonuclease 2, lysosomal [Source:HGNC Symbol;Acc:HGNC:2960]" | 1133 | 0.489 | 0.0195 | No |
| 9 | SMAP1 | small ArfGAP 1 [Source:HGNC Symbol;Acc:HGNC:19651] | 1243 | 0.468 | 0.0205 | No |
| 10 | ATP5IF1 | ATP synthase inhibitory factor subunit 1 [Source:HGNC Symbol;Acc:HGNC:871] | 1575 | 0.417 | 0.0031 | No |
| 11 | CCR2 | C-C motif chemokine receptor 2 [Source:HGNC Symbol;Acc:HGNC:1603] | 1815 | 0.382 | -0.0078 | No |
| 12 | TRAF3IP2 | TRAF3 interacting protein 2 [Source:HGNC Symbol;Acc:HGNC:1343] | 1939 | 0.368 | -0.0099 | No |
| 13 | KIT | "KIT proto-oncogene, receptor tyrosine kinase [Source:HGNC Symbol;Acc:HGNC:6342]" | 2117 | 0.348 | -0.0167 | No |
| 14 | PIK3CB | "phosphatidylinositol-4,5-bisphosphate 3-kinase catalytic subunit beta [Source:HGNC Symbol;Acc:HGNC:8976]" | 2186 | 0.340 | -0.0151 | No |
| 15 | IREB2 | iron responsive element binding protein 2 [Source:HGNC Symbol;Acc:HGNC:6115] | 2215 | 0.337 | -0.0104 | No |
| 16 | MAPK14 | mitogen-activated protein kinase 14 [Source:HGNC Symbol;Acc:HGNC:6876] | 2352 | 0.320 | -0.0145 | No |
| 17 | EPO | erythropoietin [Source:HGNC Symbol;Acc:HGNC:3415] | 2369 | 0.318 | -0.0092 | No |
| 18 | BPGM | bisphosphoglycerate mutase [Source:HGNC Symbol;Acc:HGNC:1093] | 2398 | 0.315 | -0.0050 | No |
| 19 | SOD1 | superoxide dismutase 1 [Source:HGNC Symbol;Acc:HGNC:11179] | 2413 | 0.313 | 0.0003 | No |
| 20 | MB | myoglobin [Source:HGNC Symbol;Acc:HGNC:6915] | 2445 | 0.308 | 0.0042 | No |
| 21 | NCKAP1L | NCK associated protein 1 like [Source:HGNC Symbol;Acc:HGNC:4862] | 2560 | 0.296 | 0.0013 | No |
| 22 | ISG15 | ISG15 ubiquitin like modifier [Source:HGNC Symbol;Acc:HGNC:4053] | 2821 | 0.268 | -0.0136 | No |
| 23 | XKR8 | XK related 8 [Source:HGNC Symbol;Acc:HGNC:25508] | 2884 | 0.262 | -0.0131 | No |
| 24 | INPP5D | inositol polyphosphate-5-phosphatase D [Source:HGNC Symbol;Acc:HGNC:6079] | 3085 | 0.241 | -0.0238 | No |
| 25 | PRDX1 | peroxiredoxin 1 [Source:HGNC Symbol;Acc:HGNC:9352] | 3113 | 0.239 | -0.0211 | No |
| 26 | CASP3 | caspase 3 [Source:HGNC Symbol;Acc:HGNC:1504] | 3202 | 0.230 | -0.0233 | No |
| 27 | SLC4A1 | solute carrier family 4 member 1 (Diego blood group) [Source:HGNC Symbol;Acc:HGNC:11027] | 3233 | 0.227 | -0.0210 | No |
| 28 | PLA2G10 | phospholipase A2 group X [Source:HGNC Symbol;Acc:HGNC:9029] | 3315 | 0.220 | -0.0228 | No |
| 29 | ITPKB | inositol-trisphosphate 3-kinase B [Source:HGNC Symbol;Acc:HGNC:6179] | 3467 | 0.205 | -0.0305 | No |
| 30 | ZNF16 | zinc finger protein 16 [Source:HGNC Symbol;Acc:HGNC:12947] | 3519 | 0.201 | -0.0304 | No |
| 31 | RBFOX2 | RNA binding fox-1 homolog 2 [Source:HGNC Symbol;Acc:HGNC:9906] | 3523 | 0.200 | -0.0265 | No |
| 32 | ERCC2 | "ERCC excision repair 2, TFIIH core complex helicase subunit [Source:HGNC Symbol;Acc:HGNC:3434]" | 3544 | 0.198 | -0.0240 | No |
| 33 | HOXA5 | homeobox A5 [Source:HGNC Symbol;Acc:HGNC:5106] | 4062 | 0.150 | -0.0615 | No |
| 34 | JAK2 | Janus kinase 2 [Source:HGNC Symbol;Acc:HGNC:6192] | 4183 | 0.140 | -0.0681 | No |
| 35 | TRIM58 | tripartite motif containing 58 [Source:HGNC Symbol;Acc:HGNC:24150] | 4280 | 0.130 | -0.0729 | No |
| 36 | STAT1 | signal transducer and activator of transcription 1 [Source:HGNC Symbol;Acc:HGNC:11362] | 4495 | 0.113 | -0.0874 | No |
| 37 | SH2B3 | SH2B adaptor protein 3 [Source:HGNC Symbol;Acc:HGNC:29605] | 4960 | 0.076 | -0.1222 | No |
| 38 | MERTK | "MER proto-oncogene, tyrosine kinase [Source:HGNC Symbol;Acc:HGNC:7027]" | 5085 | 0.066 | -0.1306 | No |
| 39 | FANCC | FA complementation group C [Source:HGNC Symbol;Acc:HGNC:3584] | 5138 | 0.061 | -0.1334 | No |
| 40 | HMGB2 | high mobility group box 2 [Source:HGNC Symbol;Acc:HGNC:5000] | 5272 | 0.050 | -0.1428 | No |
| 41 | FAM210B | family with sequence similarity 210 member B [Source:HGNC Symbol;Acc:HGNC:16102] | 5372 | 0.042 | -0.1497 | No |
| 42 | UBA5 | ubiquitin like modifier activating enzyme 5 [Source:HGNC Symbol;Acc:HGNC:23230] | 5390 | 0.041 | -0.1502 | No |
| 43 | TAL1 | "TAL bHLH transcription factor 1, erythroid differentiation factor [Source:HGNC Symbol;Acc:HGNC:11556]" | 5406 | 0.040 | -0.1506 | No |
| 44 | MPL | "MPL proto-oncogene, thrombopoietin receptor [Source:HGNC Symbol;Acc:HGNC:7217]" | 5508 | 0.032 | -0.1579 | No |
| 45 | STAT5B | signal transducer and activator of transcription 5B [Source:HGNC Symbol;Acc:HGNC:11367] | 5554 | 0.028 | -0.1608 | No |
| 46 | THRA | thyroid hormone receptor alpha [Source:HGNC Symbol;Acc:HGNC:11796] | 5638 | 0.021 | -0.1669 | No |
| 47 | NCAPG2 | non-SMC condensin II complex subunit G2 [Source:HGNC Symbol;Acc:HGNC:21904] | 5700 | 0.016 | -0.1714 | No |
| 48 | INHA | inhibin subunit alpha [Source:HGNC Symbol;Acc:HGNC:6065] | 5701 | 0.016 | -0.1710 | No |
| 49 | RHAG | Rh associated glycoprotein [Source:HGNC Symbol;Acc:HGNC:10006] | 5714 | 0.014 | -0.1717 | No |
| 50 | CEBPG | CCAAT enhancer binding protein gamma [Source:HGNC Symbol;Acc:HGNC:1837] | 5734 | 0.013 | -0.1729 | No |
| 51 | TMOD3 | tropomodulin 3 [Source:HGNC Symbol;Acc:HGNC:11873] | 5944 | -0.001 | -0.1893 | No |
| 52 | RPS19 | ribosomal protein S19 [Source:HGNC Symbol;Acc:HGNC:10402] | 5952 | -0.002 | -0.1898 | No |
| 53 | ACVR2A | activin A receptor type 2A [Source:HGNC Symbol;Acc:HGNC:173] | 5960 | -0.002 | -0.1903 | No |
| 54 | RB1 | RB transcriptional corepressor 1 [Source:HGNC Symbol;Acc:HGNC:9884] | 6013 | -0.006 | -0.1943 | No |
| 55 | PRMT1 | protein arginine methyltransferase 1 [Source:HGNC Symbol;Acc:HGNC:5187] | 6319 | -0.029 | -0.2176 | No |
| 56 | ACVR1B | activin A receptor type 1B [Source:HGNC Symbol;Acc:HGNC:172] | 6368 | -0.033 | -0.2207 | No |
| 57 | SLC7A11 | solute carrier family 7 member 11 [Source:HGNC Symbol;Acc:HGNC:11059] | 6470 | -0.040 | -0.2278 | No |
| 58 | LYN | "LYN proto-oncogene, Src family tyrosine kinase [Source:HGNC Symbol;Acc:HGNC:6735]" | 6514 | -0.043 | -0.2303 | No |
| 59 | SMAD5 | SMAD family member 5 [Source:HGNC Symbol;Acc:HGNC:6771] | 6530 | -0.045 | -0.2305 | No |
| 60 | CDK5RAP3 | CDK5 regulatory subunit associated protein 3 [Source:HGNC Symbol;Acc:HGNC:18673] | 6583 | -0.048 | -0.2336 | No |
| 61 | AHSP | alpha hemoglobin stabilizing protein [Source:HGNC Symbol;Acc:HGNC:18075] | 6719 | -0.057 | -0.2431 | No |
| 62 | TSPO2 | translocator protein 2 [Source:HGNC Symbol;Acc:HGNC:21256] | 6836 | -0.066 | -0.2508 | No |
| 63 | TGFBR3 | transforming growth factor beta receptor 3 [Source:HGNC Symbol;Acc:HGNC:11774] | 6868 | -0.069 | -0.2518 | No |
| 64 | SP3 | Sp3 transcription factor [Source:HGNC Symbol;Acc:HGNC:11208] | 7033 | -0.082 | -0.2630 | No |
| 65 | FOXP3 | forkhead box P3 [Source:HGNC Symbol;Acc:HGNC:6106] | 7035 | -0.082 | -0.2614 | No |
| 66 | G6PD | glucose-6-phosphate dehydrogenase [Source:HGNC Symbol;Acc:HGNC:4057] | 7082 | -0.086 | -0.2632 | No |
| 67 | CITED2 | Cbp/p300 interacting transactivator with Glu/Asp rich carboxy-terminal domain 2 [Source:HGNC Symbol;Acc:HGNC:1987] | 7100 | -0.087 | -0.2628 | No |
| 68 | PKNOX1 | PBX/knotted 1 homeobox 1 [Source:HGNC Symbol;Acc:HGNC:9022] | 7225 | -0.097 | -0.2705 | No |
| 69 | EPB42 | erythrocyte membrane protein band 4.2 [Source:HGNC Symbol;Acc:HGNC:3381] | 7228 | -0.098 | -0.2687 | No |
| 70 | UFL1 | UFM1 specific ligase 1 [Source:HGNC Symbol;Acc:HGNC:23039] | 7329 | -0.105 | -0.2744 | No |
| 71 | ETV2 | ETS variant transcription factor 2 [Source:HGNC Symbol;Acc:HGNC:3491] | 7365 | -0.108 | -0.2749 | No |
| 72 | PIK3CD | "phosphatidylinositol-4,5-bisphosphate 3-kinase catalytic subunit delta [Source:HGNC Symbol;Acc:HGNC:8977]" | 7405 | -0.111 | -0.2757 | No |
| 73 | ARID4A | AT-rich interaction domain 4A [Source:HGNC Symbol;Acc:HGNC:9885] | 7612 | -0.128 | -0.2892 | No |
| 74 | HCLS1 | hematopoietic cell-specific Lyn substrate 1 [Source:HGNC Symbol;Acc:HGNC:4844] | 7653 | -0.131 | -0.2897 | No |
| 75 | BMP4 | bone morphogenetic protein 4 [Source:HGNC Symbol;Acc:HGNC:1071] | 7816 | -0.142 | -0.2995 | No |
| 76 | HSPA1A | heat shock protein family A (Hsp70) member 1A [Source:HGNC Symbol;Acc:HGNC:5232] | 7876 | -0.146 | -0.3011 | No |
| 77 | SLC25A5 | solute carrier family 25 member 5 [Source:HGNC Symbol;Acc:HGNC:10991] | 7877 | -0.146 | -0.2981 | No |
| 78 | CDK6 | cyclin dependent kinase 6 [Source:HGNC Symbol;Acc:HGNC:1777] | 8393 | -0.185 | -0.3347 | No |
| 79 | STAT3 | signal transducer and activator of transcription 3 [Source:HGNC Symbol;Acc:HGNC:11364] | 8453 | -0.190 | -0.3355 | No |
| 80 | MPIG6B | megakaryocyte and platelet inhibitory receptor G6b [Source:HGNC Symbol;Acc:HGNC:13937] | 8471 | -0.191 | -0.3329 | No |
| 81 | LDB1 | LIM domain binding 1 [Source:HGNC Symbol;Acc:HGNC:6532] | 8522 | -0.195 | -0.3328 | No |
| 82 | AXL | AXL receptor tyrosine kinase [Source:HGNC Symbol;Acc:HGNC:905] | 8565 | -0.199 | -0.3320 | No |
| 83 | SCNN1B | sodium channel epithelial 1 subunit beta [Source:HGNC Symbol;Acc:HGNC:10600] | 8945 | -0.227 | -0.3571 | No |
| 84 | SPI1 | Spi-1 proto-oncogene [Source:HGNC Symbol;Acc:HGNC:11241] | 9012 | -0.232 | -0.3575 | No |
| 85 | RCOR1 | REST corepressor 1 [Source:HGNC Symbol;Acc:HGNC:17441] | 9120 | -0.241 | -0.3610 | No |
| 86 | SLC11A2 | solute carrier family 11 member 2 [Source:HGNC Symbol;Acc:HGNC:10908] | 9152 | -0.244 | -0.3584 | No |
| 87 | HSPA9 | heat shock protein family A (Hsp70) member 9 [Source:HGNC Symbol;Acc:HGNC:5244] | 9173 | -0.246 | -0.3549 | No |
| 88 | ADAR | adenosine deaminase RNA specific [Source:HGNC Symbol;Acc:HGNC:225] | 9195 | -0.247 | -0.3515 | No |
| 89 | DMTN | dematin actin binding protein [Source:HGNC Symbol;Acc:HGNC:3382] | 9211 | -0.249 | -0.3476 | No |
| 90 | BAK1 | BCL2 antagonist/killer 1 [Source:HGNC Symbol;Acc:HGNC:949] | 9266 | -0.253 | -0.3467 | No |
| 91 | MAFB | MAF bZIP transcription factor B [Source:HGNC Symbol;Acc:HGNC:6408] | 9320 | -0.257 | -0.3456 | No |
| 92 | BAX | "BCL2 associated X, apoptosis regulator [Source:HGNC Symbol;Acc:HGNC:959]" | 9323 | -0.257 | -0.3405 | No |
| 93 | JAM3 | junctional adhesion molecule 3 [Source:HGNC Symbol;Acc:HGNC:15532] | 9376 | -0.262 | -0.3392 | No |
| 94 | ARNT | aryl hydrocarbon receptor nuclear translocator [Source:HGNC Symbol;Acc:HGNC:700] | 9427 | -0.267 | -0.3377 | No |
| 95 | PRKDC | "protein kinase, DNA-activated, catalytic subunit [Source:HGNC Symbol;Acc:HGNC:9413]" | 9632 | -0.286 | -0.3478 | No |
| 96 | ETS1 | "ETS proto-oncogene 1, transcription factor [Source:HGNC Symbol;Acc:HGNC:3488]" | 9795 | -0.301 | -0.3543 | No |
| 97 | SLC25A38 | solute carrier family 25 member 38 [Source:HGNC Symbol;Acc:HGNC:26054] | 9904 | -0.312 | -0.3564 | No |
| 98 | BAP1 | BRCA1 associated protein 1 [Source:HGNC Symbol;Acc:HGNC:950] | 10151 | -0.336 | -0.3688 | No |
| 99 | TRIM10 | tripartite motif containing 10 [Source:HGNC Symbol;Acc:HGNC:10072] | 10169 | -0.338 | -0.3633 | No |
| 100 | ALAS2 | 5'-aminolevulinate synthase 2 [Source:HGNC Symbol;Acc:HGNC:397] | 10247 | -0.346 | -0.3622 | No |
| 101 | PTBP3 | polypyrimidine tract binding protein 3 [Source:HGNC Symbol;Acc:HGNC:10253] | 10259 | -0.347 | -0.3560 | No |
| 102 | KLF1 | Kruppel like factor 1 [Source:HGNC Symbol;Acc:HGNC:6345] | 10350 | -0.356 | -0.3558 | No |
| 103 | RPS14 | ribosomal protein S14 [Source:HGNC Symbol;Acc:HGNC:10387] | 10558 | -0.379 | -0.3642 | No |
| 104 | ANXA1 | annexin A1 [Source:HGNC Symbol;Acc:HGNC:533] | 10824 | -0.407 | -0.3767 | Yes |
| 105 | SFXN1 | sideroflexin 1 [Source:HGNC Symbol;Acc:HGNC:16085] | 10880 | -0.414 | -0.3725 | Yes |
| 106 | JAK3 | Janus kinase 3 [Source:HGNC Symbol;Acc:HGNC:6193] | 10908 | -0.418 | -0.3661 | Yes |
| 107 | GATA1 | GATA binding protein 1 [Source:HGNC Symbol;Acc:HGNC:4170] | 10958 | -0.425 | -0.3612 | Yes |
| 108 | GATA2 | GATA binding protein 2 [Source:HGNC Symbol;Acc:HGNC:4171] | 11058 | -0.439 | -0.3600 | Yes |
| 109 | MED1 | mediator complex subunit 1 [Source:HGNC Symbol;Acc:HGNC:9234] | 11146 | -0.450 | -0.3576 | Yes |
| 110 | INHBA | inhibin subunit beta A [Source:HGNC Symbol;Acc:HGNC:6066] | 11237 | -0.462 | -0.3553 | Yes |
| 111 | PTPN2 | protein tyrosine phosphatase non-receptor type 2 [Source:HGNC Symbol;Acc:HGNC:9650] | 11270 | -0.467 | -0.3482 | Yes |
| 112 | FOXO3 | forkhead box O3 [Source:HGNC Symbol;Acc:HGNC:3821] | 11305 | -0.472 | -0.3412 | Yes |
| 113 | KLF2 | Kruppel like factor 2 [Source:HGNC Symbol;Acc:HGNC:6347] | 11350 | -0.478 | -0.3349 | Yes |
| 114 | MAPK11 | mitogen-activated protein kinase 11 [Source:HGNC Symbol;Acc:HGNC:6873] | 11501 | -0.501 | -0.3364 | Yes |
| 115 | KITLG | KIT ligand [Source:HGNC Symbol;Acc:HGNC:6343] | 11516 | -0.503 | -0.3272 | Yes |
| 116 | BRD1 | bromodomain containing 1 [Source:HGNC Symbol;Acc:HGNC:1102] | 11549 | -0.507 | -0.3193 | Yes |
| 117 | KLF13 | Kruppel like factor 13 [Source:HGNC Symbol;Acc:HGNC:13672] | 11594 | -0.515 | -0.3123 | Yes |
| 118 | ZBTB7A | zinc finger and BTB domain containing 7A [Source:HGNC Symbol;Acc:HGNC:18078] | 11600 | -0.516 | -0.3021 | Yes |
| 119 | RPS6 | ribosomal protein S6 [Source:HGNC Symbol;Acc:HGNC:10429] | 11662 | -0.526 | -0.2961 | Yes |
| 120 | EPAS1 | endothelial PAS domain protein 1 [Source:HGNC Symbol;Acc:HGNC:3374] | 11758 | -0.542 | -0.2925 | Yes |
| 121 | HIF1A | hypoxia inducible factor 1 subunit alpha [Source:HGNC Symbol;Acc:HGNC:4910] | 11833 | -0.556 | -0.2869 | Yes |
| 122 | ID2 | inhibitor of DNA binding 2 [Source:HGNC Symbol;Acc:HGNC:5361] | 11852 | -0.560 | -0.2769 | Yes |
| 123 | BCL2L11 | BCL2 like 11 [Source:HGNC Symbol;Acc:HGNC:994] | 11867 | -0.564 | -0.2664 | Yes |
| 124 | ADGRF5 | adhesion G protein-coupled receptor F5 [Source:HGNC Symbol;Acc:HGNC:19030] | 11876 | -0.566 | -0.2555 | Yes |
| 125 | MFHAS1 | multifunctional ROCO family signaling regulator 1 [Source:HGNC Symbol;Acc:HGNC:16982] | 11898 | -0.571 | -0.2454 | Yes |
| 126 | HAMP | hepcidin antimicrobial peptide [Source:HGNC Symbol;Acc:HGNC:15598] | 11909 | -0.573 | -0.2345 | Yes |
| 127 | ADAM17 | ADAM metallopeptidase domain 17 [Source:HGNC Symbol;Acc:HGNC:195] | 11931 | -0.579 | -0.2243 | Yes |
| 128 | PDE4B | phosphodiesterase 4B [Source:HGNC Symbol;Acc:HGNC:8781] | 12057 | -0.609 | -0.2216 | Yes |
| 129 | ACIN1 | apoptotic chromatin condensation inducer 1 [Source:HGNC Symbol;Acc:HGNC:17066] | 12059 | -0.609 | -0.2092 | Yes |
| 130 | RPS17 | ribosomal protein S17 [Source:HGNC Symbol;Acc:HGNC:10397] | 12130 | -0.626 | -0.2019 | Yes |
| 131 | HMGB1 | high mobility group box 1 [Source:HGNC Symbol;Acc:HGNC:4983] | 12170 | -0.636 | -0.1920 | Yes |
| 132 | IKZF1 | IKAROS family zinc finger 1 [Source:HGNC Symbol;Acc:HGNC:13176] | 12186 | -0.640 | -0.1800 | Yes |
| 133 | RPS24 | ribosomal protein S24 [Source:HGNC Symbol;Acc:HGNC:10411] | 12223 | -0.652 | -0.1695 | Yes |
| 134 | GATA3 | GATA binding protein 3 [Source:HGNC Symbol;Acc:HGNC:4172] | 12276 | -0.670 | -0.1599 | Yes |
| 135 | HSPA1B | heat shock protein family A (Hsp70) member 1B [Source:HGNC Symbol;Acc:HGNC:5233] | 12278 | -0.670 | -0.1463 | Yes |
| 136 | DYRK3 | dual specificity tyrosine phosphorylation regulated kinase 3 [Source:HGNC Symbol;Acc:HGNC:3094] | 12379 | -0.702 | -0.1397 | Yes |
| 137 | HMOX1 | heme oxygenase 1 [Source:HGNC Symbol;Acc:HGNC:5013] | 12421 | -0.714 | -0.1284 | Yes |
| 138 | IL6 | interleukin 6 [Source:HGNC Symbol;Acc:HGNC:6018] | 12460 | -0.730 | -0.1164 | Yes |
| 139 | B2M | beta-2-microglobulin [Source:HGNC Symbol;Acc:HGNC:914] | 12507 | -0.750 | -0.1047 | Yes |
| 140 | SRF | serum response factor [Source:HGNC Symbol;Acc:HGNC:11291] | 12549 | -0.773 | -0.0921 | Yes |
| 141 | VEGFA | vascular endothelial growth factor A [Source:HGNC Symbol;Acc:HGNC:12680] | 12629 | -0.813 | -0.0816 | Yes |
| 142 | JMJD6 | "jumonji domain containing 6, arginine demethylase and lysine hydroxylase [Source:HGNC Symbol;Acc:HGNC:19355]" | 12658 | -0.832 | -0.0668 | Yes |
| 143 | BCL6 | BCL6 transcription repressor [Source:HGNC Symbol;Acc:HGNC:1001] | 12667 | -0.838 | -0.0503 | Yes |
| 144 | HIPK2 | homeodomain interacting protein kinase 2 [Source:HGNC Symbol;Acc:HGNC:14402] | 12842 | -1.045 | -0.0426 | Yes |
| 145 | ZFP36 | ZFP36 ring finger protein [Source:HGNC Symbol;Acc:HGNC:12862] | 12846 | -1.052 | -0.0213 | Yes |
| 146 | ZFP36L1 | ZFP36 ring finger protein like 1 [Source:HGNC Symbol;Acc:HGNC:1107] | 12888 | -1.244 | 0.0009 | Yes |
Table: GSEA details [plain text format]

  

Fig 2: GOBP\_MYELOID\_CELL\_HOMEOSTASIS      
 Blue-Pink O' Gram in the Space of the Analyzed GeneSet

  

Fig 3: GOBP\_MYELOID\_CELL\_HOMEOSTASIS: Random ES distribution      
 Gene set null distribution of ES for **GOBP\_MYELOID\_CELL\_HOMEOSTASIS**
